# Resistance, Virulence, and Molecular Epidemiology of Carbapenem-Resistant *Klebsiella pneumoniae* Causing Bloodstream Infections in Saudi Arabia

**DOI:** 10.3390/microorganisms14020333

**Published:** 2026-01-30

**Authors:** Fetoon M. Alkhelaiwi, Ali M. Somily, Reham M. Alahmadi, Maaweya Awadalla, Ahmed M. Albarrag, Bandar Alosaimi, Eman Marzouk, Ihab M. Moussa

**Affiliations:** 1 Department of Botany and Microbiology, College of Science, King Saud University, Riyadh 11451, Saudi Arabia; falklewiy@ksu.edu.sa (F.M.A.); realahmadi@ksu.edu.sa (R.M.A.); imoussa1@ksu.edu.sa (I.M.M.); 2 Department of Pathology, College of Medicine, King Saud University and King Saud University Medical City, Riyadh 11461, Saudi Arabia; somily@ksu.edu.sa (A.M.S.);; 3 Biomedical Administration, Research Center, King Fahad Medical City, Riyadh Second Health Cluster, Riyadh 11525, Saudi Arabia; 4 Department of Public Health, College of Applied Medical Sciences, Qassim University, Buraydah 51452, Saudi Arabia

**Keywords:** *Klebsiella pneumoniae*, carbapenem resistance, bloodstream infection, whole-genome sequencing, OXA-48-like, NDM, clonal dissemination

## Abstract

Carbapenem-resistant *Klebsiella pneumoniae* (CRKP) has become a major cause of bloodstream infections and poses serious challenges to clinical management because treatment options are limited. This study aimed to characterize antimicrobial resistance, virulence-associated features, and molecular epidemiology of CRKP bloodstream isolates using integrated phenotypic and genomic approaches. A total of 74 non-duplicate CRKP isolates were collected from bloodstream infections at three tertiary-care hospitals in Riyadh, Saudi Arabia, between 2022 and 2024. All isolates showed classical *Klebsiella pneumoniae* phenotypic characteristics, including intrinsic resistance to natural and aminopenicillins, and were classified as either multidrug-resistant (MDR) or extensively drug-resistant (XDR). Resistance to imipenem was universal, and resistance to other β-lactams and fluoroquinolones was high. Carbapenemase genes were detected in 96.0% of isolates using the GeneXpert^®^ Carba-R assay, with *bla*_OXA-48-like_ and *bla*_NDM_ being most common. Whole-genome sequencing demonstrated predominance of Ambler class D carbapenemases, particularly *bla*_OXA-232_, with additional contributions from *bla*_NDM-1_ and *bla*_NDM-5_. Co-occurrence of carbapenemase genes was observed in a subset of isolates. Virulence analysis showed that 37.8% of isolates exhibited a hypermucoviscous phenotype, and more than half carried at least one virulence-associated determinant linked to capsule regulation or iron acquisition. In contrast, most isolates showed weak or no biofilm-forming capacity. Multilocus sequence typing revealed substantial genetic diversity but clear dominance of high-risk lineages, particularly ST147 and the emerging ST2096, both closely associated with *bla*_OXA-232_ and *bla*_OXA-48-like_ genes. Capsular and O-antigen analysis showed a non-random distribution dominated by KL64 and O1/O2. Phylogenetic analysis was consistent with clonal expansion and suggested intra-hospital spread, with the intensive care unit serving as a key reservoir and dissemination to other wards. In conclusion, CRKP bloodstream infections in this setting are largely associated with a limited number of epidemic clones that combine extensive antimicrobial resistance with virulence-associated traits. These findings support the need for ongoing genome-based surveillance, strengthened infection control measures, and antimicrobial stewardship to limit the spread of high-risk *K. pneumoniae* lineages in healthcare settings.

## 1. Introduction

Antimicrobial resistance (AMR) is one of the most serious global threats to public health and continues to undermine effective treatment of bacterial infections. The rising prevalence of resistant pathogens has resulted in prolonged hospital stays, increased healthcare costs, and higher morbidity and mortality worldwide [1,2,3,4]. Excessive and inappropriate use of antimicrobial agents in healthcare and community settings has intensified selective pressure on bacterial populations. Additional contributors include antibiotic use in livestock, inadequate infection prevention practices, and horizontal transfer of resistance genes mediated by mobile genetic elements [5,6,7].

In recognition of this growing challenge, the World Health Organization has identified AMR as a major global health priority and has emphasized the need for strengthened surveillance and coordinated international action [8,9]. The COVID-19 pandemic further amplified the AMR burden, as increased antibiotic prescribing and prolonged intensive care unit (ICU) admissions created favorable conditions for the emergence and spread of resistant organisms [10,11]. In response, initiatives such as the Global Antimicrobial Resistance Surveillance System were established to improve monitoring and support evidence-based interventions [12].

Among antimicrobial-resistant pathogens, members of the ESKAPE group (*Enterococcus faecium*, *Staphylococcus aureus*, *Klebsiella pneumoniae*, *Acinetobacter baumannii*, *Pseudomonas aeruginosa*, and *Enterobacter* spp.) are of particular concern because of their ability to evade antimicrobial therapy and cause severe healthcare-associated infections [9,13]. Within this group, *K. pneumoniae* represents a major clinical and epidemiological challenge. This opportunistic Gram-negative bacterium is a leading cause of pneumonia, urinary tract infections, and bloodstream infections, especially among critically ill and immunocompromised patients [14,15,16]. Its ability to colonize mucosal surfaces, persist in hospital environments, and acquire resistance determinants facilitates its widespread dissemination in healthcare settings.

The global expansion of carbapenem-resistant *K. pneumoniae* (CRKP) has raised serious concerns regarding treatment failure and adverse patient outcomes. Carbapenems are commonly reserved as last-line agents for severe infections caused by multidrug-resistant Gram-negative bacteria. Resistance to these agents is therefore associated with limited therapeutic options and high mortality, which often exceeds 40% in bloodstream infections [17,18]. In *K. pneumoniae*, carbapenem resistance is mainly driven by carbapenemase production, including *bla*_KPC_, *bla*_NDM_, and *bla*_OXA-48-like_ variants. These mechanisms frequently act alongside porin alterations and efflux systems, resulting in high-level resistance under antimicrobial pressure.

In the Middle East, and particularly in Saudi Arabia, CRKP has become an important public health concern. Surveillance studies have reported increasing rates of carbapenemase-producing Enterobacterales, with prevalence estimates ranging from 10% to 30% [19]. OXA-48-like and NDM-type carbapenemases predominate in the region and reflect epidemiological patterns that differ from those observed in Europe and North America [20]. The growing detection of resistance to last-resort agents such as colistin and tigecycline further complicates clinical management and highlights the need for detailed molecular investigations [21].

In addition to antimicrobial resistance, increasing attention has been directed toward virulence-associated features of CRKP. Classical *K. pneumoniae* strains linked to healthcare-associated infections were traditionally considered to have limited virulence, whereas hypervirulent strains were mainly associated with community-acquired invasive disease. Recent reports have described the increasing detection of virulence-associated genes among carbapenem-resistant strains, suggesting convergence of resistance and virulence traits. These organisms may cause severe infections, including bloodstream infections, even in patients without classical risk factors, which raises concern for public health [22].

Understanding the molecular epidemiology of CRKP requires integrated analysis of resistance mechanisms, virulence-associated determinants, and clonal relationships. Whole-genome sequencing (WGS) and multilocus sequence typing (MLST) have become essential tools for identifying high-risk lineages, exploring genetic relatedness, and clarifying the basis of resistance and pathogenicity. Such approaches are particularly valuable in regions with high CRKP prevalence and limited genomic surveillance data.

Despite the increasing burden of CRKP in Saudi Arabia, comprehensive studies that integrate phenotypic resistance profiles, resistance gene characterization, virulence-associated determinants, and clonal relatedness of bloodstream isolates remain limited. This gap restricts the ability to assess dissemination patterns, recognize emerging high-risk clones, and guide targeted infection control strategies. Therefore, the present study aims to provide an integrated phenotypic and genomic characterization of CRKP causing bloodstream infections in Riyadh, Saudi Arabia. By analyzing antimicrobial susceptibility patterns, carbapenemase genes, virulence-associated determinants, efflux pump profiles, and clonal relationships, this work seeks to clarify the epidemiological landscape of CRKP and identify emerging threats relevant to both regional and global healthcare settings.

## 2. Materials and Methods

### 2.1. Ethical Approval

This study was approved by the Institutional Review Board of King Saud University, Riyadh, Saudi Arabia (Protocol No. E-23-7629/IRB). The study was conducted in accordance with institutional and national research guidelines. Because of the retrospective laboratory-based design and use of non-identifiable clinical isolates, informed consent was waived.

### 2.2. Study Design and Bacterial Isolates

This descriptive study included clinical isolates recovered from hospitalized patients at King Khalid University Hospital (KKUH), King Fahd Medical City (KFMC), and King Saud Medical City (KSMC) in Riyadh, Saudi Arabia. Between 2022 and 2024, a total of 74 non-duplicate CRKP isolates were obtained from bloodstream infections. Only one isolate per patient was included. Isolates were categorized according to patient demographics, infection source, antimicrobial susceptibility profiles, and sequence types.

Bloodstream infections were defined based on microbiological confirmation from positive blood cultures obtained during hospitalization. Due to the retrospective, laboratory-based nature of the study and the absence of standardized clinical timelines, infections could not be consistently classified as primary or secondary bloodstream infections, nor could they be definitively categorized as community- or hospital-acquired.

### 2.3. Bacterial Identification and Antimicrobial Susceptibility Testing

All isolates were recovered from blood cultures processed in the clinical microbiology laboratories of the participating hospitals. Identification as *K. pneumoniae* was performed using automated systems, including VITEK^®^2 (bioMérieux, Durham, NC, USA) and Phoenix™ BD (Becton Dickinson Diagnostic Systems, Sparks, MD, USA), according to the manufacturers’ instructions.

Antimicrobial susceptibility testing was conducted using the same automated platforms and confirmed when required using E-test strips (bioMérieux, Durham, NC, USA). Results were interpreted according to Clinical and Laboratory Standards Institute (CLSI) guidelines [23]. Isolates were classified as carbapenem-resistant based on resistance to at least one carbapenem agent.

### 2.4. Phenotypic Characterization

#### 2.4.1. Mucoid Phenotype and Hypermucoviscosity

Mucoid phenotype and hypermucoviscosity were assessed using the string test. Isolates were cultured on MacConkey agar and incubated overnight at 37 °C. A standard inoculation loop was used to stretch a viscous string from a single colony. Formation of a string measuring at least 5 mm was considered a positive result, as previously described [24].

#### 2.4.2. Biofilm Formation Assay

Biofilm formation was evaluated using a microtiter plate assay, as previously described [25,26]. Assays were performed in triplicate using 96-well flat-bottom plates. Bacterial suspensions were adjusted to a 0.5 McFarland standard in tryptic soy broth and incubated under static conditions at 37 °C for 48 h. Biofilms were stained with crystal violet, solubilized with ethanol, and optical density was measured at 570 nm. Isolates were classified as non, weak, moderate, or strong biofilm producers based on optical density values relative to negative controls.

### 2.5. DNA Extraction and Quality Assessment

Genomic DNA was extracted from overnight cultures using the QIAamp DNA Mini Kit (Qiagen, Hilden, Germany) following the manufacturer’s instructions. DNA concentration and purity were assessed using a NanoDrop™ 2000 spectrophotometer (Thermo Scientific, Waltham, MA, USA). Samples with A260/A280 ratios between 1.8 and 2.0 were considered suitable for downstream analyses.

### 2.6. Molecular Detection of Carbapenemase Genes

Carbapenemase genes were detected using the automated real-time PCR-based GeneXpert^®^ Carba-R assay on the GeneXpert IV system (Cepheid, Sunnyvale, CA, USA), according to the manufacturer’s protocol. The assay targets *bla*_KPC_, *bla*_IMP_, *bla*_NDM_, *bla*_OXA-48-like_, and *bla*_VIM_. Results were automatically interpreted within one hour, as previously described [27].

### 2.7. PCR Detection of Capsular and Virulence-Associated Genes

Multiplex and duplex PCR assays were used to detect capsular serotype-specific and virulence-associated genes. Primer sets targeting capsular types (K1, K2, K3, K5, K20, K54, and K57) and virulence-associated genes (*rmpA*, *fimH*, *entB*, *fepA*, *iucA*, *iroN*, *iroD*, *kfuBC*, *uge*, *wcaG*, and *alls*) were obtained commercially (Macrogen, Seoul, Republic of Korea) and applied as previously described [28].

PCR amplification was performed using a GeneAmp^®^ 9700 thermal cycler (Applied Biosystems, Melbourne, Australia) with FIREPol^®^ Master Mix (Solis BioDyne, Tartu, Estonia). Cycling conditions consisted of an initial denaturation at 95 °C for 5 min, followed by 30 cycles of denaturation at 95 °C for 30 s, annealing at 57 °C for 60 s, and extension at 72 °C for 1 min, with a final extension at 72 °C for 10 min. Primer sequences and amplicon sizes are provided in Table S1.

### 2.8. Agarose Gel Electrophoresis

PCR products were analyzed by 1% agarose gel electrophoresis using 1× TAE buffer, as described by Sambrook [29]. Gels were stained with SYBR^®^ Safe DNA Gel Stain and visualized under ultraviolet illumination. DNA fragment sizes were estimated using 100 bp and 1 kb DNA ladders (Qiagen, Hilden, Germany; Thermo Fisher Scientific, Waltham, MA, USA).

### 2.9. Whole-Genome Sequencing and Library Preparation

WGS libraries were prepared using the QIAGEN^®^ QIAseq FX DNA Library Kit (QIAGEN, Hilden, Germany) following the manufacturer’s instructions. Library preparation included enzymatic fragmentation, end repair, adapter ligation, amplification, and purification. Library quality and fragment size distribution were assessed using the Agilent 4200 TapeStation system with high-sensitivity D1000 reagents. Libraries with the expected size distribution were used for downstream sequencing.

### 2.10. Bioinformatic and Phylogenetic Analysis

Sequencing data were analyzed using the Bactopia bioinformatics pipeline. The workflow included quality control, genome assembly, annotation, resistance gene detection, MLST assignment, and phylogenetic analysis. Core-genome phylogenies were inferred using pan-genome analysis with Roary without requiring a reference genome. Phylogenetic relationships were examined to assess clonal relatedness among isolates.

## 3. Results

### 3.1. Phenotypic Analysis

All *K. pneumoniae* isolates produced large, smooth, convex, mucoid lactose-fermenting colonies on MacConkey agar and were non-hemolytic on blood agar. Microscopy confirmed Gram-negative, non-motile bacilli. A total of 74 non-duplicate CRKP isolates from bloodstream infections were included in the analysis.

CRKP bloodstream infections were detected across all age groups, with the highest prevalence among patients aged ≥65 years (36.8%), followed by those aged 46–65 years (26.5%). Males accounted for 62.0% of cases.

### 3.2. Demographic Characteristics of Patients

Patients were stratified into five age groups: 0–18, 19–25, 26–45, 46–65, and ≥65 years. CRKP isolates were detected across all age categories, with a clear predominance among older patients. Individuals aged ≥65 years accounted for the largest proportion of cases (36.8%), followed by those aged 46–65 years (26.5%) and 26–45 years (23.5%). Younger age groups were less frequently affected, with patients aged 19–25 years and 0–18 years accounting for 7.4% and 5.9% of cases, respectively. The youngest patient included in the study was 57 days old, while the oldest patient was 95 years old. Overall, CRKP bloodstream infections were more common in male patients (62.0%) than in female patients (38.0%).

### 3.3. Distribution of Clinical Ward Types and Specimen Sources

CRKP bloodstream isolates were recovered from both ICU and non-ICU wards. Non-ICU locations accounted for 54.0% (40/74) of cases, while ICU settings accounted for 46.0% (34/74). Most isolates were obtained from central venous line-associated blood cultures (70.3%), whereas peripheral blood cultures represented 29.7% of specimens. Central venous lines were the predominant source of CRKP bloodstream infections in both ICU and non-ICU settings.

### 3.4. AMR Profiles and Temporal Trends

AMR profiles of CRKP bloodstream isolates collected between 2022 and 2024 are summarized in Table S2. Temporal trends in resistance are shown in Figure S1. AST was performed using VITEK^®^2 or Phoenix™ BD systems, and results were interpreted according to CLSI guidelines. Resistance rates are reported as percentages for each antimicrobial agent by year and for the overall study period.

Across all three years, CRKP isolates showed uniformly high resistance to β-lactam antibiotics, including aminopenicillin–β-lactamase inhibitor combinations, cephalosporins, and carbapenems. Resistance to imipenem was observed in all isolates throughout the study period. Resistance to meropenem and ertapenem also remained high, with only minor year-to-year variation (Table S2). These findings indicate a stable carbapenem-resistant phenotype among circulating CRKP isolates.

Substantial resistance was also observed among non–β-lactam agents. Resistance to fluoroquinolones was consistently high, with ciprofloxacin and levofloxacin showing elevated resistance rates across all years. Aminoglycoside resistance varied, with gentamicin exhibiting higher resistance rates than amikacin. Resistance to trimethoprim sulfamethoxazole was common and remained relatively stable during the study period (Table S2).

Analysis of temporal trends revealed modest year-to-year variation in resistance patterns (Figure S1). Resistance to most antimicrobial classes remained largely unchanged between 2022 and 2024. In contrast, resistance to colistin and tigecycline showed a gradual increase over time, although overall resistance rates to these agents remained lower than those observed for other antibiotics. The highest overall resistance burden was observed in 2023, corresponding to the peak in CRKP isolation during the study period.

Overall, these results demonstrate persistent multidrug resistance among CRKP bloodstream isolates, characterized by sustained resistance to carbapenems and multiple non β-lactam agents, with limited evidence of susceptibility recovery over time.

### 3.5. Virulence-Associated Phenotypes and Biofilm Formation

Among the 74 CRKP bloodstream isolates, 28 isolates (37.8%) exhibited a hypermucoviscous phenotype based on a positive string test and were classified as hypermucoviscous *K. pneumoniae*. This phenotype is commonly associated with increased capsule production.

To further assess virulence-associated features, isolates were screened for genes related to iron acquisition, capsule regulation, and other virulence-associated functions. Overall, 41 isolates (55.4%) carried at least one gene previously linked to hypervirulence-associated lineages (Table 1). Genes involved in iron acquisition were highly prevalent. The enterobactin biosynthesis gene *entB* was detected in 94.6% of isolates, and the adhesin-encoding gene *fimH* was identified in 92.0%. Aerobactin-associated genes (*iucA*, *iucD*, and *iutA*) and yersiniabactin-associated genes (*fyuA* and *ybt*) were detected at moderate to high frequencies. Capsule-associated regulatory genes, including *rmpA* and *rmpA2*, were also identified, indicating the presence of virulence-associated genetic determinants among a substantial subset of isolates.

Biofilm-forming capacity was assessed using a microtiter plate assay. In contrast to the frequent detection of hypermucoviscosity and virulence-associated genes, most CRKP isolates showed limited biofilm formation. Specifically, 64 isolates (86.5%) were classified as non-biofilm producers, while 10 isolates (13.5%) exhibited weak biofilm formation. No isolates demonstrated moderate or strong biofilm-forming capacity.

These findings indicate a virulence-associated profile characterized by frequent hypermucoviscosity and widespread carriage of genes linked to capsule function and siderophore-mediated iron acquisition, alongside a generally low capacity for biofilm formation among CRKP bloodstream isolates.

### 3.6. Molecular Detection of Carbapenemase Genes Using the GeneXpert^®^ Carba-R Assay

Carbapenemase genes were detected using the GeneXpert^®^ Carba-R assay in 71 of the 74 CRKP bloodstream isolates, corresponding to an overall positivity rate of 96.0% (Table 2). The most frequently detected determinant was *bla*_OXA-48-like_, identified in 39 isolates (52.7%). The *bla*_NDM_ gene was detected in 12 isolates (16.2%), while *bla*_KPC_ and *bla*_VIM_ were identified in 3 (4.05%) and 1 (1.35%) isolates, respectively. No isolates carried *bla*IMP.

Co-occurrence of carbapenemase genes was observed in a subset of isolates. The combination of *bla*_OXA-48-like_ and *bla*_NDM_ was detected in 15 isolates (20.27%). One isolate (1.35%) harbored both *bla*_KPC_ and *bla*_NDM_. Three isolates (4.05%) tested negative for all carbapenemase genes included in the assay. All GeneXpert^®^ Carba-R assay results were generated within 32–48 min.

### 3.7. Classification of Carbapenemase Genes Identified by Whole-Genome Analysis

WGS-based analysis was performed to characterize carbapenemase resistance determinants among the 74 CRKP bloodstream isolates. Resistance genes were identified using assembled genomic data analyzed through the Bactopia pipeline with curated resistance databases.

Carbapenemase genes showed an uneven distribution across Ambler classes (Figure S2). Class D carbapenemases were the predominant resistance mechanism and were detected in 54 isolates (73.0%). Within this class, *bla*_OXA-232_ was the most frequent determinant, identified in 34 isolates (46.0%), followed by *bla*_OXA-48_ in 17 isolates (23.0%) and *bla*_OXA-181_ in 3 isolates (4.0%).

Class B metallo-β-lactamases were detected in 29 isolates (39.2%). The most common genes were *bla*_NDM-1_, identified in 15 isolates (20.27%), and *bla*_NDM-5_, detected in 13 isolates (17.56%). The *bla*_VIM-1_ gene was rare and identified in a single isolate (1.35%).

Class A carbapenemases were infrequently detected. The *bla*_KPC-2_ gene was identified in four isolates, accounting for 5.4% of the total study population. Three isolates (4.0%) were negative for all screened carbapenemase genes and were classified as carbapenemase negative.

Overall, WGS data demonstrated a clear predominance of OXA-type carbapenemases among CRKP bloodstream isolates, with *bla*_OXA-232_ representing the most frequent determinant. A substantial contribution from NDM-type metallo-β-lactamases was also observed. The distribution of carbapenemase genes by Ambler class is summarized in Figure S2.

### 3.8. Temporal Distribution of Carbapenemase Genes During the Study Period

Analysis of temporal trends revealed that 2023 represented the peak year for carbapenemase gene detection among CRKP bloodstream isolates. This increase was primarily driven by the higher prevalence of *bla*_OXA-232_ and NDM-type carbapenemases.

Overall, carbapenemase distribution was dominated by Ambler Class D enzymes, which were detected in the majority of isolates throughout the study period. Among these, *bla*_OXA-232_ was the most prevalent determinant and was consistently identified across all three years. Class B metallo-β-lactamases, particularly *bla*_NDM-1_ and *bla*_NDM-5_, showed marked temporal clustering in 2023, with only sporadic detections in 2022 and 2024. In contrast, Class A carbapenemases (*bla*_KPC-2_) were infrequently detected and restricted to a single year, indicating limited and non-sustained circulation.

Co-occurrence of carbapenemase classes was observed in a subset of isolates, most commonly involving concurrent detection of Class B and Class D enzymes. This pattern highlights the ability of *K. pneumoniae* to accumulate multiple resistance determinants. Carbapenemase-negative isolates were uncommon and evenly distributed across the study period. Detailed year-by-year distributions and classification of carbapenemase genes are provided in Table 3.

### 3.9. Efflux Pump Genes

Multiple efflux pump–associated genes were detected among the CRKP bloodstream isolates. Core efflux components were highly conserved. The genes *acrB*, *acrD*, and *msbA* were detected in all 74 isolates, indicating universal presence of these systems. The *oqxA* gene was identified in 73 isolates (98.65%), while *oqxB* was detected in 70 isolates (94.60%), reflecting widespread distribution of the OqxAB efflux system.

In contrast, several auxiliary efflux-related genes were detected at low frequencies. The genes *acrE*, *acrF*, *emrA*, *emrB*, *tolC*, and *voiJ* were each identified in four isolates (5.4%). Overall, these findings indicate broad conservation of major efflux systems among CRKP isolates, with limited dissemination of additional efflux pump components.

### 3.10. MLST Analysis

Multilocus sequence typing revealed substantial clonal diversity among the *K. pneumoniae* bloodstream isolates, with 22 distinct sequence types identified. Despite this diversity, a limited number of high-risk clones predominated. Sequence types ST147 and ST2096 together accounted for approximately half of all analyzed isolates, highlighting their central role in the dissemination of carbapenem resistance in this setting. The remaining isolates were distributed among multiple less frequent sequence types, each contributing only a small proportion of the total population. The overall distribution of MLST sequence types is illustrated in Figure S3.

### 3.11. Capsular and O-Antigen Diversity Among K. pneumoniae Sequence Types

Given the predominance of specific MLST lineages, capsular (K-locus) and O-antigen (O-locus) diversity was further examined (Table 4). A total of fourteen distinct capsular loci were identified among the 74 CRKP bloodstream isolates. The KL64 locus was the most prevalent capsular type and was detected in 47 isolates (63.51%). Other capsular loci were identified at substantially lower frequencies. Six isolates (8.1%) could not be assigned to a known K-locus.

Analysis of O-antigen biosynthesis loci revealed a non-random distribution dominated by O1 and O2 serotypes, which were collectively identified in 61 isolates (82.43%). The O4 locus was detected in five isolates (6.75%), while O3b and O5 were each identified in a single isolate. Two isolates carried unassigned O-loci.

A clear association between sequence type and capsular locus was observed. The dominant sequence types ST147 and ST2096 were uniformly associated with the KL64 capsular locus and predominantly carried O1 or O2 antigens. In contrast, less frequent sequence types exhibited greater heterogeneity in both K- and O-locus composition.

### 3.12. Correlation of MLST with Carbapenem-Resistance Genes

Correlation analysis between MLST and carbapenem-resistance genes demonstrated a strong linkage between dominant clonal lineages and specific carbapenemase determinants (Table 5). ST147 displayed the highest degree of genetic diversity and was associated with multiple carbapenemase genes, including *bla*_OXA-232_, *bla*_NDM-1_, *bla*_OXA-48_, and *bla*_NDM-5_, either alone or in combination.

ST2096 showed a highly uniform resistance profile, with all isolates carrying *bla*OXA-232. A similar exclusive association with *bla*_OXA-232_ was observed for ST14. ST11 was characterized by consistent carriage of *bla*_KPC-2_, with occasional detection of *bla*_NDM-1_. ST231 demonstrated a mixed resistance profile, encoding either *bla*_NDM-5_ or *bla*_OXA-232_.

Less frequent sequence types generally showed one-to-one associations with single carbapenemase genes. Two rare sequence types, ST1655 and ST985, were negative for all screened carbapenemase genes. These findings indicate that carbapenem resistance within this cohort is largely structured by clonal lineage. High-risk sequence types, particularly ST147 and ST2096, are closely associated with specific carbapenemase determinants and dominant capsular and O-antigen configurations, reflecting clonal expansion of epidemiologically successful CRKP lineages.

### 3.13. Distribution of Carbapenem-Resistant Genes Across Hospital Wards

To examine the clinical distribution of carbapenem resistance, carbapenemase genes were analyzed according to MLST sequence type, year of isolation, and hospital ward (Table 6). Carbapenemase genes were unevenly distributed across intensive care, medical, surgical, and emergency settings and were predominantly associated with high-risk clonal lineages.

The ICU accounted for the largest proportion of carbapenem-resistant isolates, particularly those belonging to ST147 and ST2096. Medical wards represented the second most frequent source and contributed substantially to dissemination of *bla*_OXA-232_ and *bla*_OXA-48_, with occasional detection of *bla*_NDM_ and *bla*_KPC_ genes. Surgical wards accounted for fewer isolates and included strains carrying *bla*_OXA-232_ and *bla*_NDM-5_. Isolates recovered from the emergency department were sporadic, suggesting either early detection or limited onward spread.

Temporal and spatial analysis showed that most carbapenemase gene detections occurred in 2023, particularly within ICU and medical wards. Notably, *bla***_VIM-1_** was detected exclusively in the ICU in 2023, indicating sporadic emergence of less common resistance mechanisms. Phenotypically, most isolates demonstrated high-level resistance to imipenem and meropenem, with an MIC_50_ of 32 µg/mL. These findings highlight the predominance of OXA-type carbapenemases, especially *bla***_OXA-232_**, within high-risk lineages such as ST147 and ST2096 and identify the ICU as a key setting for the emergence and dissemination of CRKP in the hospital environment.

### 3.14. Phylogenetic Analysis of CRKP

**Figure 1:**
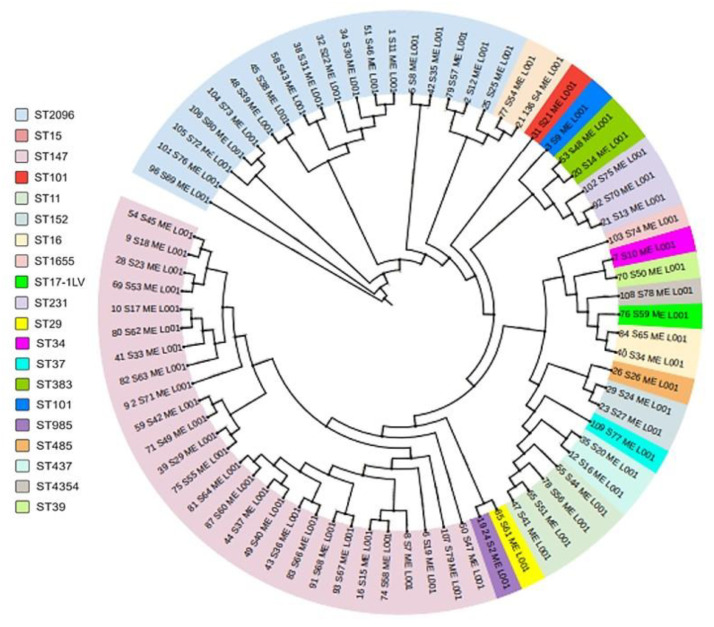
Core-genome phylogenetic analysis of CRKP bloodstream isolates. A circular phylogenetic tree constructed from WGS data of 74 *K. pneumoniae* isolates shows clustering by MLST sequence type. Branches are colored by sequence type, illustrating population structure and clonal relationships. Dominant lineages, particularly ST147 and ST2096, form distinct clusters, while less frequent sequence types appear as small clusters or singletons.

To explore the genetic relatedness among CRKP bloodstream isolates, a core-genome phylogenetic analysis was performed using WGS data. The resulting phylogeny revealed a structured population composed of multiple distinct clades that largely corresponded to MLST sequence types and highlighted the dominance of a limited number of high-risk lineages (Figure 1).

The phylogenetic tree showed clear clustering of isolates belonging to ST147 and ST2096, supporting clonal expansion of these lineages within the study population. These sequence types formed well-defined branches and accounted for the majority of CRKP isolates. Their clustering was consistent with their widespread detection across ICU and non-ICU wards and their frequent association with *bla*_OXA-232_, *bla*_OXA-48_, and *bla*_NDM_-type carbapenemases. The close genetic relatedness observed within each lineage suggests sustained local spread rather than repeated independent introductions.

Other established healthcare-associated lineages, including ST11 and ST231, formed separate clusters but remained phylogenetically related to the dominant ST147 and ST2096 clades. ST11 isolates carrying *bla*KPC-2 grouped within a distinct subclade, indicating genetic separation between KPC-producing strains and the predominant OXA-type carbapenemase–producing lineages in this cohort.

Less frequent sequence types, including ST29, ST34, ST37, ST39, ST152, ST383, ST437, ST485, ST1655, and ST4354, were distributed as single isolates or small clusters throughout the phylogeny. These isolates typically carried a single carbapenemase gene, such as *bla*_VIM-1_, *bla*_OXA-181_, or *bla*_NDM-1_, and showed limited evidence of broader dissemination.

In general, the phylogenetic structure underscores the central role of ST147 and ST2096 as dominant epidemic lineages associated with carbapenem resistance in this setting. The observed clustering patterns support ongoing clonal expansion and emphasize the value of genome-based surveillance for monitoring transmission dynamics and guiding infection control strategies.

## 4. Discussion

CRKP has emerged as a major cause of bloodstream infections and represents a growing challenge for clinical care worldwide. Bloodstream infections caused by CRKP are associated with high mortality and limited treatment options, particularly in hospital settings with sustained antimicrobial pressure. Despite this clinical impact, integrated data linking resistance mechanisms, virulence-associated traits, and clonal structure of invasive CRKP remain limited in regions where *OXA-48*-like and *NDM* carbapenemases predominate. In this context, the present study provides a combined phenotypic, molecular, and genomic analysis of CRKP bloodstream isolates from tertiary-care hospitals, offering clinically relevant insight into the dissemination of high-risk clones in healthcare environments.

At the phenotypic level, all CRKP bloodstream isolates displayed classical characteristics, including mucoid lactose-fermenting colonies, non-hemolytic growth, and Gram-negative bacillary morphology. These findings are consistent with established descriptions of clinical *K. pneumoniae* and confirm the reliability of routine culture-based identification [30,31]. While these phenotypic features support routine laboratory diagnosis, they cannot be used alone to infer virulence potential.

CRKP bloodstream infections affected all age groups but were most frequent among older adults, particularly patients aged ≥65 years. This distribution is consistent with previous reports identifying advanced age as a major risk factor for CRKP infection [30,32]. Increased susceptibility in this population is commonly associated with healthcare exposure, comorbid conditions, invasive procedures, and antimicrobial use. Detection of CRKP in neonates and children, although uncommon, confirms susceptibility in younger populations in settings with healthcare-associated transmission [33]. Male predominance likely reflects differences in healthcare exposure rather than inherent biological susceptibility.

CRKP infections occurred in both ICU and non-ICU wards, with a slightly higher proportion detected outside the ICU. Most isolates were recovered from central venous line–associated blood cultures, identifying intravascular devices as a major source of infection. This finding is consistent with reports describing ICUs as key reservoirs for CRKP due to high antimicrobial pressure, while also highlighting dissemination beyond critical care settings [34]. Central venous catheters remain well-recognized risk factors for CRKP bacteremia, particularly when infection prevention practices are suboptimal [35]. Differences in ward distribution reported elsewhere likely reflect institutional practices and patient populations [36].

Antimicrobial susceptibility testing revealed persistently high resistance to β-lactams, including carbapenems, along with substantial resistance to fluoroquinolones and aminoglycosides. Resistance to colistin and tigecycline remained low but showed a gradual increase. These patterns are consistent with global surveillance data and reflect sustained antimicrobial pressure in hospital settings [37,38]. Even modest increases in resistance to last-resort agents are clinically concerning, as they threaten remaining therapeutic options. Regional differences likely reflect variation in antimicrobial use and stewardship practices [39].

Virulence analysis showed that 37.8% of isolates exhibited a hypermucoviscous phenotype, and more than half carried at least one hypervirulence-associated gene. However, hypermucoviscosity should be interpreted cautiously, as phenotype–genotype discordance is well documented [40,41,42]. The high prevalence of *entB* and *fimH* supports their conserved roles in iron acquisition and adhesion [43,44,45]. Detection of aerobactin-, yersiniabactin-, and capsule-associated regulatory genes supports increasing reports of resistance–virulence convergence in CRKP [46,47,48]. In contrast, most isolates showed weak or absent biofilm formation, suggesting that invasive potential in this cohort may rely more on capsule- and siderophore-mediated mechanisms than on biofilm-associated persistence [49,50].

Molecular screening identified carbapenemase genes in 96% of isolates, with predominance of *bla*_OXA-48-like_ genes followed by *bla*_NDM_. Lower frequencies of *bla*_KPC_ and *bla*_VIM_ were observed, and *bla*_IMP_ was not detected. This distribution reflects regional and global CRKP epidemiology [51,52]. The high detection rate supports carbapenemase production as the principal mechanism of carbapenem resistance in this cohort. Co-occurrence of carbapenemase genes, particularly *bla*_OXA-48-like_ with *bla*_NDM_, highlights the capacity of *K. pneumoniae* to accumulate multiple resistance determinants [53]. The rapid turnaround of the GeneXpert^®^ Carba-R assay supports its value for early detection and infection control interventions [54].

WGS analysis confirmed a predominance of Ambler class D carbapenemases, mainly *bla*_OXA-232_, with a substantial contribution from NDM variants. This pattern aligns with reports from the Gulf region and global surveillance identifying OXA-48-like and NDM enzymes as major drivers of CRKP dissemination [52,55,56,57]. In contrast, other regions report higher prevalence of class A carbapenemases, particularly *bla_KPC_*, highlighting geographic heterogeneity [58,59]. Variation in dominant OXA-48-like subtypes reported elsewhere further reflects local clonal and plasmid dynamics [57,60]. The small proportion of carbapenemase-negative isolates likely reflects alternative resistance mechanisms, including porin alterations and other β-lactamases [61,62].

Marked year-to-year variation was observed, with 2023 representing a peak in carbapenemase detection driven mainly by *bla_OXA-232_* and NDM variants. Similar temporal patterns linked to clonal expansion and hospital transmission have been described in longitudinal studies [63,64,65]. Restriction of *bla_KPC-__2_* to a single year suggests limited introduction without sustained establishment [66,67].

Efflux analysis demonstrated near-universal conservation of core systems, including *acrB*, *acrD*, and *msbA*, confirming their role as a resistance backbone in *K. pneumoniae* [68,69,70]. The widespread presence of the OqxAB system further supports its association with multidrug resistance, whereas auxiliary efflux genes were infrequent and strain-specific [71,72,73].

MLST analysis revealed substantial genetic diversity but clear dominance of ST147 and ST2096. ST147 is a recognized high-risk clone frequently associated with OXA-48-like and NDM carbapenemases and healthcare-associated outbreaks [74,75,76]. ST2096, an emerging lineage in the Middle East, showed a strong association with *bla_OXA-__232_* [20,77]. Other sequence types were detected sporadically, suggesting repeated introductions rather than sustained transmission.

Capsular and O-antigen analysis demonstrated strong linkage between dominant clones and specific antigenic profiles, particularly KL64 combined with O1 or O2 among ST147 and ST2096, consistent with global data linking capsule type to clonal success and invasive disease [78,79,80].

Ward-based analysis identified the ICU as the primary reservoir for CRKP, with spread into medical wards. This distribution reflects antimicrobial pressure, invasive procedures, and patient movement within hospitals [81,82]. Phylogenetic analysis confirmed tight clustering of ST147 and ST2096, supporting ongoing intra-hospital transmission rather than repeated importation [77,83].

In summary, CRKP bloodstream infections in this setting are driven mainly by clonal expansion of high-risk lineages, particularly ST147 and ST2096, carrying OXA-48-like and NDM carbapenemases. The convergence of resistance, virulence-associated traits, and successful clonal backgrounds underscores the clinical and epidemiological threat posed by CRKP. Integration of phenotypic, molecular, and genomic data highlights the importance of genome-based surveillance to guide infection control strategies and limit dissemination in healthcare settings.

## 5. Limitations of the Study

This study has several limitations that should be considered when interpreting the findings. First, the retrospective, laboratory-based design limited access to detailed clinical data. Information on patient comorbidities, severity of illness, prior antimicrobial exposure, treatment regimens, clinical outcomes, and mortality was not systematically available. As a result, direct correlations between resistance or virulence profiles and clinical outcomes could not be assessed.

In addition, the retrospective design and limited availability of detailed clinical timelines precluded systematic differentiation between primary and secondary bloodstream infections. It also prevented definitive classification of infections as community- or hospital-acquired. Routine surveillance or control swabs for colonization were not systematically performed prior to bloodstream infection diagnosis. Therefore, baseline colonization status and the possibility of prior carriage could not be assessed.

Second, although isolates were collected from three major tertiary-care hospitals, the study was geographically confined to a single city. Therefore, the findings may not fully reflect the molecular epidemiology of CRKP bloodstream infections in other regions of Saudi Arabia or neighboring countries, where antimicrobial use, infection control practices, and circulating lineages may differ.

Third, the sample size was modest and limited to bloodstream isolates. While this focus enhances relevance to invasive infections, it restricts extrapolation to other clinical syndromes, such as respiratory, urinary tract, or intra-abdominal infections, where resistance and virulence patterns may differ.

Fourth, hypervirulence was evaluated using phenotypic assessment and detection of virulence-associated genes, but in vivo virulence assays or animal infection models were not performed. Consequently, although the presence of hypervirulence-associated determinants suggests increased pathogenic potential, virulence could not be conclusively established based on genetic data alone.

Fifth, the analysis focused on gene presence rather than gene expression. Resistance mechanisms such as efflux pump overexpression, porin alterations, and regulatory pathway activation may contribute to carbapenem resistance beyond simple gene carriage. Without transcriptomic or proteomic analyses, the functional contribution of these mechanisms could not be quantified.

Sixth, long-read sequencing and plasmid reconstruction were not performed. This limited detailed characterization of the genetic context, mobility, and transmission dynamics of carbapenemase and virulence genes. As a result, it was not possible to definitively distinguish between clonal expansion and plasmid-mediated dissemination as the dominant mode of spread.

Finally, although phylogenetic analysis supported clonal expansion and intra-hospital transmission, environmental sampling and healthcare worker screening were not included. This precluded identification of potential environmental reservoirs or transmission pathways contributing to the persistence of high-risk clones.

Despite these limitations, the study provides an integrated analysis combining phenotypic testing, molecular diagnostics, and whole-genome sequencing. The findings offer valuable insight into the epidemiology of CRKP bloodstream infections and provide a strong foundation for future prospective, multicenter, and outcome-linked genomic surveillance studies.

## 6. Conclusions

This study offers a clear picture of CRKP bloodstream infections by combining phenotypic findings with molecular and genomic data. In this setting, disease is driven mainly by a small number of high-risk clonal lineages, especially ST147 and ST2096, which are closely linked to OXA-48-like and NDM carbapenemases and are widely present across hospital wards. The high frequency of carbapenemase genes, together with resistance to multiple antimicrobial classes, leaves few therapeutic options for invasive infections and reinforces the clinical challenge posed by CRKP. Many isolates also carried virulence-associated determinants and showed hypermucoviscosity, yet biofilm formation was generally weak, suggesting that bloodstream invasion relies more on capsule expression and iron acquisition than on biofilm persistence. Phylogenetic analysis points to ongoing clonal expansion and intra-hospital spread, with the ICU acting as an important reservoir and a source for dissemination to other wards. Taken together, these findings underline the importance of genome-based surveillance, combined with strong infection control and antimicrobial stewardship, to limit the spread of high-risk CRKP lineages and protect the remaining treatment options for severe bloodstream infections.

## Figures and Tables

**Table 1:** Virulence-associated genes detected among CRKP bloodstream isolates (N = 74).

Genes	No. of Isolates (n = 74)	%	Genes	No. of Isolates (n = 74)	%
*entB*	70	94.60%	*rmpA2* **	31	41.90%
*fimH*	68	92%	*rmpA* *	26	35.13%
*fyuA*	66	89.18%	*Kfu* *	21	28.37%
*iucA* *	41	55.40%	*fepA*	20	27%
*iucD* **	41	55.40%	*alls*	12	16.21%
*uge*	38	51.35%	*peg-344* **	9	12%
*ybt* **	38	51.35%	*rmpC* **	8	11%
*iutA* *	35	47%	*rmpD* **	7	10%

* Hypervirulence-associated genes. ** Genes associated with hypervirulence and identified using whole-genome sequencing data.

**Table 2:** Distribution of carbapenemase genes among CRKP bloodstream isolates detected using the GeneXpert^®^ Carba-R assay (N = 74).

Xpert Carba-R Assay Result	2022	2023	2024	Specimens (n = 74)	%
Positive	8	55	8	71	96.0
*bla*_KPC_	0	3	0	3	4.05
*bla*_IMP_	0	0	0	0	0.0
*bla*_NDM_	0	10	2	12	16.22
*bla*_VIM_	0	1	0	1	1.35
*bla*_OXA-48-like_	6	27	6	39	52.70
*bla*_KPC_ + *bla*_NDM_	0	1	0	1	1.35
*bla*_OXA-48-like_ + *bla*_NDM_	2	13	0	15	20.27
Negative	1	1	1	3	4.05

**Table 3:** Temporal distribution of carbapenemase genes detected among CRKP bloodstream isolates from 2022 to 2024.

Ambler Class	Carbapenemase Gene	2022	2023	2024	Total Isolates (N)	% of Total Isolates
Class A (N = 4)	*bla*_KPC-2_	0	4	0	4	5.4
Class B (N = 29)	*bla*_NDM-1_	1	14	0	15	20.27
	*bla*_NDM-5_	1	11	1	13	17.56
	*bla*_VIM-1_	0	1	0	1	1.35
Class D (N = 54)	*bla*_OXA-232_	4	26	4	34	46.0
	*bla*_OXA-48_	3	12	2	17	23.0
	*bla*_OXA-181_	0	2	1	3	4.0
Other (N = 3)	Negative	1	1	1	3	4.0

**Table 4:** Distribution of capsular (K-locus) and O-antigen (O-locus) types among *K. pneumoniae* sequence types and their associated carbapenemase genes.

ST	Isolate Count	Most Common K-Type	O-Type (O-Loci)	Detected Carbapenemase Genes
ST147	27	KL64	O2a	*bla*_NDM-1_; *bla*_NDM-5_; *bla*_OXA-232_; *bla*_OXA-48_
ST2096	18	KL64	O1	*bla*_OXA-232_
ST11	4	KL47	unknown (OL101)	*bla*_KPC-2_; *bla*_NDM-1_
ST231	3	KL51	O1	*bla*_NDM-5_; *bla*_OXA-232_
ST14	2	KL64	O1	*bla*_OXA-232_
ST437	2	KL36	O4	*bla*_NDM-5_; *bla*_OXA-232_
ST152	2	KL149	O4	*bla*_NDM-1_; *bla*_OXA-48_
ST383	2	KL30	O1	*bla*_NDM-5_; *bla*_OXA-48_
ST34	1	KL119	O4	*bla*_VIM-1_
ST485	1	KL28	O2a	*bla*_OXA-48_
ST4354	1	KL9	O2afg	*bla*_OXA-48_
ST39	1	KL62	O1	*bla*_OXA-48_
ST37	1	KL14	O3b	*bla*_OXA-181_
ST101	1	KL17	O1	*bla*_OXA-48_
ST29	1	KL24	O1	*bla*_NDM-1_
ST17-1LV	1	KL25	O5	*bla*_NDM-1_
ST1655	1	KL158	O1	-
ST16-1LV	1	KL51	O2afg	*bla*_OXA-181_
ST16	1	KL81	unknown (OL101)	*bla*_NDM-5_; *bla*_OXA-181_
ST15	1	KL112	O1	*bla*_NDM-1_
ST147-1LV	1	KL64	O2a	*bla*_OXA-48_
ST985	1	KL39	O1	-

**Table 5:** Correlation of MLST sequence types with carbapenemase genes detected among CRKP bloodstream isolates (N = 74).

MLST (N)	*bla*_NDM-1_	*bla*_NDM-5_	*bla*_OXA-232_	*bla*_OXA-48_	*bla*_OXA-181_	*bla*_KPC-2_	*bla*_VIM-1_	NEG
ST147 (27)	10 (37.0%)	6 (22.2%)	12 (44.4%)	9 (33.3%)	-	-	-	-
ST2096 (18)	-	-	18 (100%)	-	-	-	-	-
ST11 (4)	1 (25%)	-	-	-	-	4 (100%)	-	-
ST231 (3)	-	2 (66.7%)	1 (33.3%)	-	-	-	-	1 (33.3%)
ST383 (2)	-	2 (100%)	-	2 (100%)	-	-	-	-
ST437 (2)	-	2 (100%)	1 (50%)	-	-	-	-	-
ST152 (2)	1 (50%)	-	-	1 (50%)	-	-	-	-
ST14 (2)	-	-	2 (100%)	-	-	-	-	-
ST101 (1)	-	-	-	1 (100%)	-	-	-	-
ST147-1LV (1)	-	-	-	1 (100%)	-	-	-	-
ST15 (1)	1 (100%)	-	-	-	-	-	-	-
ST16 (1)	-	1 (100%)	-	-	1 (100%)	-	-	-
ST16-1LV (1)	-	-	-	-	1 (100%)	-	-	-
ST17-1LV (1)	1 (100%)	-	-	-	-	-	-	-
ST29 (1)	1 (100%)	-	-	-	-	-	-	-
ST34 (1)	-	-	-	-	-	-	1 (100%)	-
ST37 (1)	-	-	-	-	1 (100%)	-	-	-
ST39 (1)	-	-	-	1 (100%)	-	-	-	-
ST4354 (1)	-	-	-	1 (100%)	-	-	-	-
ST485 (1)	-	-	-	1 (100%)	-	-	-	-
ST1655 (1)	-	-	-	-	-	-	-	1 (100%)
ST985 (1)	-	-	-	-	-	-	-	1 (100%)

**Table 6:** Distribution of carbapenem-resistant genes among CRKP bloodstream isolates according to MLST sequence type, year of isolation, and hospital ward (2022–2024).

MLST	Gene	2022	2023	2024	Location	MLST	Gene	2022	2023	2024	Location
ST147 N = 27	*bla*_NDM-1_	0	10	0	4 ICU/MED/SURG/4 ER	ST147-1LV	*bla*_OXA-48_	0	1	0	MED
	*bla*_OXA-232_	0	12	0	5 ICU/2 MED/2 SURG/3 ER	ST15 N = 1	*bla*_NDM-1_	0	1	0	ICU
	*bla*_OXA-48_	2	7	0	4 ICU/2 MED/2 SURG/ER	ST16 N = 1	*bla*_NDM-5_	0	1	0	ICU
	*bla*_NDM-5_	0	5	1	3 ICU/MED/SURG/ER	ST17-1LV N = 1	*bla*_OXA-181_	0	1	0	ICU
ST2096 N = 18	*bla*_OXA-232_	3	11	4	7 ICU/5 MED/3 SURG/3 ER	ST16-1LV N = 1	*bla*_OXA-181_	0	1	0	ER
ST11 N = 4	*bla*_KPC-2_	0	4	0	2 ICU/MED/ER	ST17-1LV N = 1	*bla*_NDM-1_	0	1	0	ICU
	*bla*_NDM-1_	0	1	0	MED	ST29 N = 1	*bla*_NDM-1_	0	0	1	ICU
ST231 N = 3	*bla*_NDM-5_	0	2	0	ICU/SURG	ST34 N = 1	*bla*_VIM-1_	0	1	0	ICU
	*bla*_OXA-232_	0	1	0	SURG	ST37 N = 1	*bla*_OXA-181_	0	0	1	ICU
	NEG	0	1	0	MED	ST39 N = 1	*bla*_OXA-48_	0	1	0	MED
ST383 N = 2	*bla*_NDM-5_	1	1	0	ER/MED	ST4354 N = 1	*bla*_OXA-48_	0	0	1	ICU
	*bla*_OXA-48_	1	1	0	ER/MED	ST485 N = 1	*bla*_OXA-48_	1	0	0	ICU
ST437 N = 2	*bla*_NDM-5_	0	2	0	ER/MED	ST1655 N = 1 ST985 N = 1	*bla*_NEG_	0	0	1	ICU
	*bla*_OXA-232_	0	1	0	ER		*bla*_NEG_	1	0	0	MED
ST152 N = 2	*bla*_NDM-1_	0	1	0	ER	ST14 N = 2	*bla*_OXA-232_	1	1	0	ICU/GS
	*bla*_OXA-48_	0	1	0	SURG	ST101 N = 1	*bla*_OXA-48_	0	1	0	ICU

## Data Availability

The original contributions presented in this study are included in the article/Supplementary Material. Further inquiries can be directed to the corresponding author.

## Supplementary Materials

The following supporting information can be downloaded at: https://www.mdpi.com/article/10.3390/microorganisms14020333/s1, Figure S1: Temporal trends in AMR among CRKP bloodstream isolates from 2022 to 2024, showing year-to-year changes in resistance patterns across selected antimicrobial agents; Figure S2: Distribution of carbapenemase genes among *K. pneumoniae* bloodstream isolates according to Ambler classification. Stacked bars represent the percentage of total isolates (N = 74) carrying individual carbapenemase genes within each Ambler class; Figure S3: Distribution of MLST sequence types among CRKP bloodstream isolates (N = 74). Horizontal bars represent the percentage contribution of each sequence type to the total isolate population; Table S1: Primer sequences for targeting capsular-encoding and virulence-associated-encoding genes; Table S2: Antimicrobial susceptibility profiles of CRKP bloodstream isolates collected between 2022 and 2024. Refs. [84,85,86,87,88,89,90,91,92,93] can be found in Supplementary Materials.
