# Supplementary material for: Resistance, Virulence, and Molecular Epidemiology of Carbapenem-Resistant Klebsiella pneumoniae Causing Bloodstream Infections in Saudi Arabia
Source: Microorganisms. 2026 Jan 30;14(2):333. doi: 10.3390/microorganisms14020333 (PMC12943425; doi:10.3390/microorganisms14020333)
Supplement: Supplementary file 1 [file microorganisms-14-00333-s001.zip › microorganisms-4121591-supplementary.pdf]

Table S1. Primer sequences for targeting capsular-encoding and virulence-associated-encoding genes.

Gene	Primer	Primer Sequence (5′---3′)	Amplicon size (bp)	function	annealing temperature	Reference
*wzy*K1	F<br>R	GGTGCTCTTTACATCATTGC<br>GCAATGGCCATTTGCGTTAG	1283	capsular serotype	57°C	[1]
*wzy*K3	F<br>R	TAGGCAATTGACTTTAGGTG<br>AGTGAATCAGCCTTCACCT	549			[2]
*wca*G	E<br>R	GGTTGGKTCAGCAATCGTA<br>ACTATTCCGCCAACTTTTGC	169	capsule biosynthesis		[3]
*wzy*K20	F<br>R	CGGTGCTACAGTGCATCATT<br>GTTATACGATGCTCAGTCGC	741	capsular serotype	59°C	[4]
*wzy*K54	F<br>R	CATTAGCTCAGTGGTTGGCT<br>GCTTGACAAACACCATAGCAG	881			
*wzy*K57	F<br>R	CTCAGGGCTAGAAGTGTCAT<br>CACTAACCCAGAAAGTCGAG	1037			[5]
fepA	F<br>R	CCTGCCGATTGATTTCCTCG<br>CGTTACGATGGTGCTGTCAG	226	iron acquisition (enterobactin)	58°C	[6]
iucA	F<br>R	ATCCACCAGCAGGTTTTCAC<br>ATCCACCAGCAGGTTTTCAC	591	iron acquisition (aerobactin)		
alls	F<br>R	CCGAAACATTACGCACCTTT<br>ATCACGAAGAGCCAGGTCAC	508	nitrogen metabolism		[7]
entB	F<br>R	ATTTCCTCAACTTCTGGGGC<br>AGCATCGGTGGCGGTGGTCA	371	iron acquisition (enterobactin)	59°C	[8]
alls	F<br>R	CCGAAACATTACGCACCTTT<br>ATCACGAAGAGCCAGGTCAC	508	nitrogen metabolism		[9]
iutA	F<br>R	GTTGCGATTCTACCCGTTCC<br>GCCCGGTGGTGTAAATCTTC	248	iron acquisition (aerobactin)	56°C	[6]
kfuBC	F<br>R	GAAGTGACGCTGTTTCTGGC<br>TTTCGTGTGGCCAGTGACTC	797	ferric iron uptake		[9]
UGE	F<br>R	TCTTCACGCCTTCCTTCACT<br>GATCATCCGGTCTCCCTGTA	534	capsular polysaccharide biosynthesis		
fimH	F<br>R	TGCTGCTGGGCTGGTCGATG<br>GGGAGGGTGACGGTGACAT	688	type 1 fimbrial adhesin	60°C	[8]
entB	F<br>R	ATTTCCTCAACTTCTGGGGC<br>AGCATCGGTGGCGGTGGTCA	371	iron acquisition (enterobactin)		
*wzy*K2	F<br>R	GACCCGATATTCATACTTGACAGAG<br>CCTGAAGTAAAATCGTAAATAGATGGC	641	capsular serotype	59°C	[1]
*wzy*K5	F<br>R	TGGTAGTGATGCTCGCGA<br>CCTGAACCCACCCCAATC	280			
fyuA	F<br>R	ATATGGCAAAAGCGCTCAGG<br>GGGTTAATCATGTCGCCGTC	204	iron acquisition (yersiniabactin)	58°C	[6]
rmpA	F<br>R	ACTGGGCTACCTCTGCTTCA<br>CTTGCATGAGCCATCTTTCA	516	Regulator of mucoid phenotype A		[10]
iroD	F<br>R	GCATAGGCGGATACGAACAT<br>CACAGGGCAATTGCTTACCT	556	iron acquisition(salmochelin)	58°C	[6]
iroN	F<br>R	GGCTACTGATACTTGACTATTC<br>CAGGATACAATAGCCCATAG	992			

References for Table S1

1. Turton, J.F.; Baklan, H.; Siu, L.; Kaufmann, M.E.; Pitt, T.L. Evaluation of a multiplex PCR for detection of serotypes K1, K2 and K5 in Klebsiella sp. and comparison of isolates within these serotypes. *FEMS microbiology letters* **2008**, *284*, 247-252.
2. Fevre, C.; Passet, V.; Deletoile, A.; Barbe, V.; Frangeul, L.; Almeida, A.S.; Sansonetti, P.; Tournebize, R.; Brisse, S. PCR-based identification of Klebsiella pneumoniae subsp. rhinoscleromatis, the agent of rhinoscleroma. *PLoS neglected tropical diseases* **2011**, *5*, e1052.
3. Jian-Li, W.; Yuan-Yuan, S.; Shou-Yu, G.; Fei-Fei, D.; Jia-Yu, Y.; Xue-Hua, W.; Yong-Feng, Z.; Shi-Jin, J.; Zhi-Jing, X. Serotype and virulence genes of Klebsiella pneumoniae isolated from mink and its pathogenesis in mice and mink. *Scientific reports* **2017**, *7*, 17291.
4. Fang, C.-T.; Lai, S.-Y.; Yi, W.-C.; Hsueh, P.-R.; Liu, K.-L.; Chang, S.-C. Klebsiella pneumoniae genotype K1: an emerging pathogen that causes septic ocular or central nervous system complications from pyogenic liver abscess. *Clinical infectious diseases* **2007**, *45*, 284-293.
5. Pan, Y.-J.; Fang, H.-C.; Yang, H.-C.; Lin, T.-L.; Hsieh, P.-F.; Tsai, F.-C.; Keynan, Y.; Wang, J.-T. Capsular polysaccharide synthesis regions in Klebsiella pneumoniae serotype K57 and a new capsular serotype. *Journal of clinical microbiology* **2008**, *46*, 2231-2240.
6. Zhao, Y.; Zhang, X.; Torres, V.V.L.; Liu, H.; Rocker, A.; Zhang, Y.; Wang, J.; Chen, L.; Bi, W.; Lin, J. An outbreak of carbapenem-resistant and hypervirulent Klebsiella pneumoniae in an intensive care unit of a major teaching hospital in Wenzhou, China. *Frontiers in public health* **2019**, *7*, 229.
7. Wu, X.; Shi, Q.; Shen, S.; Huang, C.; Wu, H. Clinical and bacterial characteristics of Klebsiella pneumoniae affecting 30-day mortality in patients with bloodstream infection. *Frontiers in cellular and infection microbiology* **2021**, *11*, 688989.
8. El Fertas-Aissani, R.; Messai, Y.; Alouache, S.; Bakour, R. Virulence profiles and antibiotic susceptibility patterns of Klebsiella pneumoniae strains isolated from different clinical specimens. *Pathologie Biologie* **2013**, *61*, 209-216.
9. Wu, X.; Liu, J.; Feng, J.; Shabbir, M.A.B.; Feng, Y.; Guo, R.; Zhou, M.; Hou, S.; Wang, G.; Hao, H. Epidemiology, environmental risks, virulence, and resistance determinants of Klebsiella pneumoniae from dairy cows in Hubei, China. *Frontiers in microbiology* **2022**, *13*, 858799.
10. Nadasy, K.A.; Domiati-Saad, R.; Tribble, M.A. Invasive klebsiella pneumoniae syndrome in North America. *Clinical Infectious Diseases* **2007**, *45*, e25-e28.

Table S2. Antimicrobial susceptibility profiles of CRKP bloodstream isolates collected between 2022 and 2024 (N = 74).

Class / Family	Antibiotics	2022	Resistance rate	2023	Resistance rate	2024	Resistance rate	Total 2022-2024	
		N=9	%	N=56	%	N=9	%	N=74	%
**Aminopenicillin + β-lactamase inhibitor**	Amoxicillin-Clavulanate	9	100	56	100	9	100	74	100
**Cephalosporins**	Cefazolin	9	100	56	100	9	100	74	100
	Cefuroxime	9	100	56	100	9	100	74	100
	Ceftriaxone	9	100	56	100	9	100	74	100
	Ceftazidime	9	100	56	100	9	100	74	100
	Cefepime	9	100	56	100	9	100	74	100
**Fluoroquinolones**	Ciprofloxacin	9	100	55	98.21	8	88.8	72	94.3
	Levofloxacin	8	88.8	52	92.85	7	77.7	67	90.5

**Aminoglycosides**	Gentamicin	5	55.5	38	67.85	7	77.7	50	67.5
	Amikacin	4	66.6	39	69.64	6	66.6	49	66.21
**sulfonamide + DHFR inhibitor**	Trimethoprim - Sulfamethoxazole	7	77.7	45	80.35	8	88.8	60	81
**Ureidopenicillin + β-lactamase inhibitor**	Piperacillin-Tazobactam	8	88.8	56	100	9	100	73	98.64
**Carbapenems**	Imipenem	9	100	56	100	9	100	74	100
	Meropenem	9	100	55	98.21	8	88.8	72	97.30
	Ertapenem	8	88.8	54	96.42	9	100	71	96
**others**	Tigecycline	0	0	3	4	1	11.1	4	5.4
	Nitrofurantoin	7	77.7	49	87.5	8	88.8	64	86.5
	Colistin	1	11.1	9	16.1	3	33.3	13	17.56

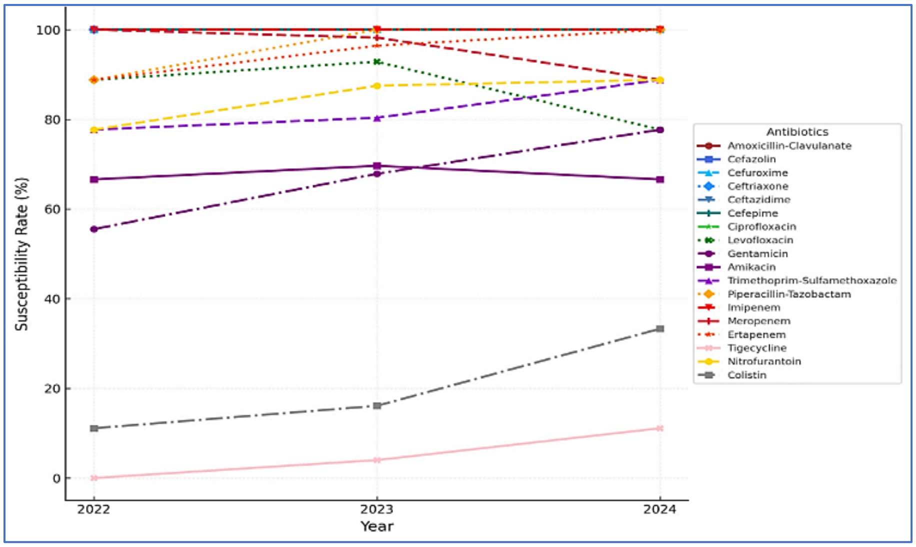

Figure S1. Temporal trends in AMR among CRKP bloodstream isolates from 2022 to 2024, showing year-to-year changes in resistance patterns across selected antimicrobial agents.

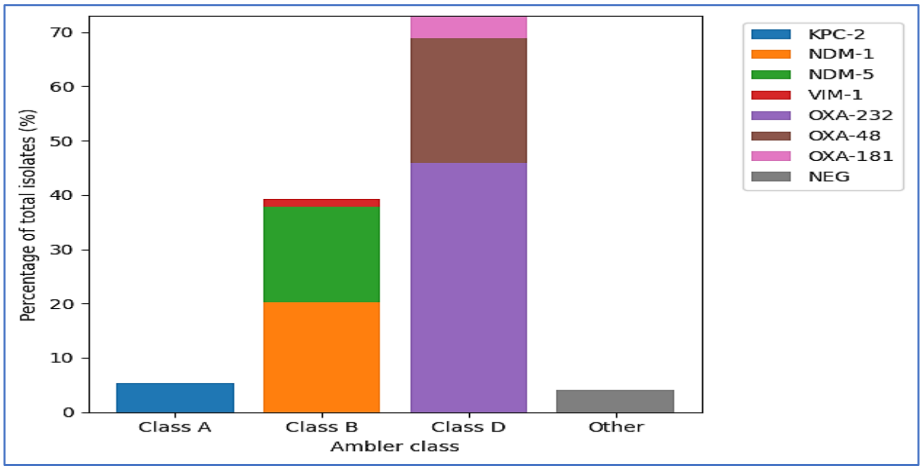

Figure S2. Distribution of carbapenemase genes among *K. pneumoniae* bloodstream isolates according to Ambler classification. Stacked bars represent the percentage of total isolates (N = 74) carrying individual carbapenemase genes within each Ambler class.

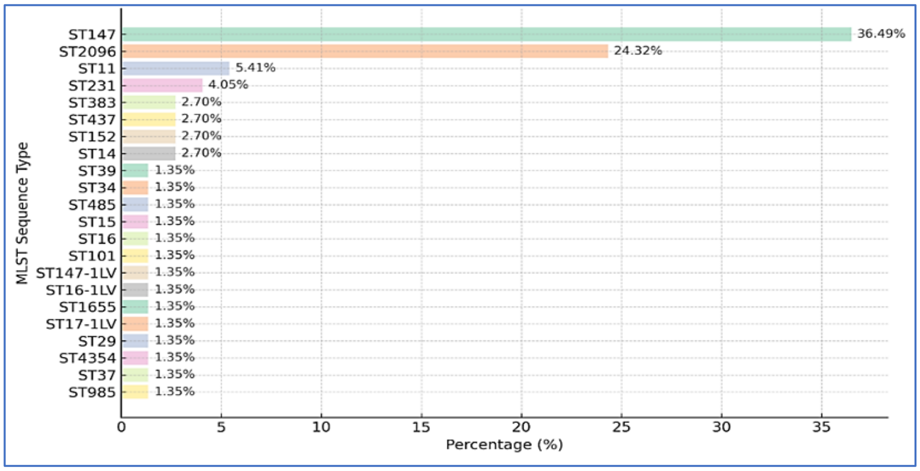

Figure S3. Distribution of MLST sequence types among CRKP bloodstream isolates (N = 74). Horizontal bars represent the percentage contribution of each sequence type to the total isolate population.
